# Sustaining the nursing workforce in ageing health systems: evidence from trend in entries, exits and capacity projections in Czechia

**DOI:** 10.1186/s13690-026-01965-5

**Published:** 2026-05-23

**Authors:** Simona Jíchová, Tereza Patáková, Luděk Šídlo

**Affiliations:** Department of Demography and Geodemography, Faculty of Science, Charles University, Czechia Albertov 6, Prague, 12800 Czechia

**Keywords:** Nurses, Workforce, Age structure, Healthcare system, Ageing, Model projection

## Abstract

**Background:**

The ageing and shortage of healthcare workers represents a major problem in many countries worldwide, including Czechia. This study focuses specifically on nurses in the Czech healthcare system. The aims of the article are to describe the age and geographical structures of nurses and analyse their entry into and exit from the healthcare system, as well as to provide projections aimed at highlighting the potential risks of a shortage of nurses.

**Methods:**

This retrospective observational study utilised data from the largest Czech health insurance company (the General Health Insurance Company of the Czech Republic (GHIC)), which has concluded contracts with almost all healthcare providers in Czechia, for the period from 31 December 2012 to 30 September 2023. Demographic methods were used to evaluate the age structures and entry and exit patterns, which was followed by the compilation of a “what-if” projection model of nursing capacities up to 2035.

**Results:**

The analysis revealed a clear ageing trend across all the country’s healthcare segments, as reflected in the increasing proportion of nurses in older age groups and the declining representation of younger age groups. If current entry and exit trends continue, projections for 2035 predict declines in both the full-time equivalent capacity (FTE) and physical capacity (headcounts) of up to 8% compared to the year 2022.

**Conclusions:**

The study points to the need for targeted planning, improved entry conditions for nursing graduates and support for personnel retention, especially in regions and care sectors most at risks of shortages. It further highlights the importance of implementing policies that ensure the long-term sustainability of the nursing workforce in an ageing healthcare system.

**Table:** 

Text box 1. Contributions to the literature	
• The ageing of the nursing workforce in a European health system (Czechia) is accelerating and, if current trends persist, will lead to significant capacity shortages within the next decade, threatening access to care.	
• This study introduces a replicable projection model using anonymised administrative workforce data, offering a practical tool for health workforce planning in systems with similar data infrastructure.	
• It reveals marked regional and specialization-specific disparities in ageing patterns, highlighting the need for targeted recruitment and retention interventions where shortages are most likely to occur.	

## Background

The issue of ageing populations is currently being subjected to intense discussion worldwide; since it affects many areas of human society, it has become a major research topic [1, 2]. Ageing populations give rise to a range of challenges, including the need to adapt healthcare systems accordingly. The increasing proportion of older people in the population, who require relatively more care than the younger population [3], is accompanied by the need for increased healthcare facility capacities and more healthcare workers, i.e. doctors, nurses and other professionals.

Along with ageing populations, healthcare workers are also ageing, thus exacerbating the problem of the existing and future predicted shortage of such professionals [4–7]. Estimates of the global demand for, and supply of, healthcare paint a negative picture in most countries globally [7]. Upper-middle-income countries are particularly vulnerable, as demographic and economic growth, combined with ageing populations, exert significant pressure on their healthcare systems.

A central policy concern for developed countries is to ensure the sufficient generational renewal of the healthcare workforce, especially nurses [8, 9]. Nurses represent a key component of the workforce and make up the largest professional group of workers in healthcare systems, and who contribute significantly to the provision of quality healthcare services. However, current studies indicate that the level of student interest in this field is declining in OECD countries. This trend is driven mainly by heavy workloads, inadequate financial remuneration and low professional prestige, all of which were exacerbated by the COVID-19 pandemic [10]. The result is a significant shortage of nurses in many countries – in 2022, 15 EU countries reported a shortage of nurses (e.g. Greece, Lithuania, Bulgaria). The EU average in the same year was 8.4 nurses per 1,000 inhabitants [11].

The number of nurses in Czechia is slightly above the European average (9.0 nurses per 1,000 inhabitants) [11]. Nevertheless, the country is experiencing shortages, the seriousness of which varies from region to region [5, 12]. However, Czechia stands out in terms of the fact that most nurses fall into the 55+ age group, while a very small proportion of nurses are under the age of 35 [13].

**Fig. 1:**
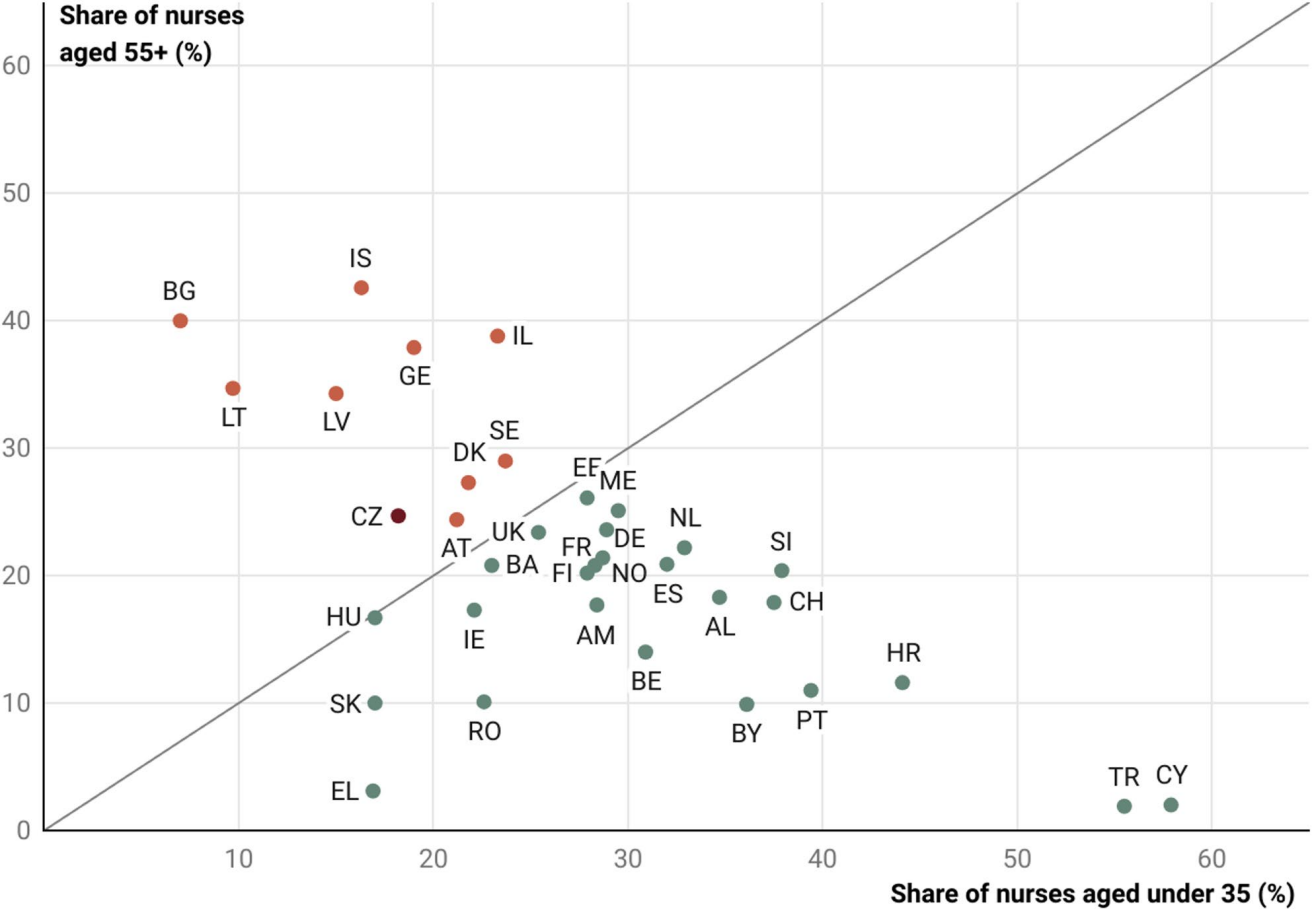
Share of nurses aged 55+ and under 35, selected European countries, 2021 (or latest data available) (%). Source: [13]; author’s own calculations. Note: AM – Armenia, AT – Austria, BA – Bosnia and Herzegovina, BE – Belgium, BG – Bulgaria, BY – Belarus, CY – Cyprus, CZ – Czech Republic, DE – Germany, DK – Denmark, EE – Estonia, EL – Greece, ES – Spain, FI – Finland, FR – France, GE – Georgia, HR – Croatia, HU – Hungary, CH – Switzerland, IE – Ireland, IS – Iceland, IL – Israel, LV – Latvia, LT – Lithuania, ME – Montenegro, NL – Netherlands, NO – Norway, PT – Portugal, RO – Romania, SE – Sweden, SI – Slovenia, SK – Slovakia, TR – Turkey, UK – United Kingdom

Similar age structures have been observed in other European countries, such as Bulgaria, Latvia, Lithuania and Iceland (Fig. 1). The main problem with significant imbalances between the numbers of older and younger nurses is that as older nurses retire, the rate of replacement by younger nurses is insufficient. Therefore, unless significant changes are implemented in terms of healthcare workforce planning, increases in the shortfall of nurses are practically inevitable. In contrast, e.g. Turkey and Cyprus have very young nurse age structures. However, it is important to note that national statistics may be significantly influenced by the quality of the data available. Major discrepancies often arise from inconsistent definitions of the nursing profession, particularly the distinction between professional nurses and nursing associate professionals [14].

Despite the alarming proportion of nurses approaching retirement age, insufficient attention has been devoted in Czechia to estimating future trends in their numbers and outlining the associated problems. The “Competent Nurse of the 21st Century” project, which analysed the education and performance of general nurses, proposed a methodology for estimating the number of students needed in general nursing education programmes [15]. The methodology responds to the “Nursing Concept” compiled by the Ministry of Health, which points to the shortage of non-medical professionals [16]. The SYRI study [17] addressed the impact of the nursing study workload on professional turnover and recommended the introduction of intervention measures aimed at improving the working and educational conditions of nursing students in Czechia.

Efforts to increase the number of nurses that enter the healthcare system in Czechia are currently being made with the recent approval of a programme that aims to increase the capacity of university education in nursing specialisations by at least 20% [18] in order to ensure the generational renewal of nurses and thus prevent a shortage of professionals.

However, none of the related studies conducted to date have focused in detail on regional differences in nursing capacities in Czechia, despite its being a particularly important issue. The ageing of healthcare workers and the increasing shortage thereof are generally most evident in rural areas [19]. For example, an Australian study showed that although rural nurses are older than their urban counterparts, they retire at higher ages [20]. Another study found that the density of nurses and midwives in Nordic countries tended to be higher in less densely populated areas, while in Czechia, the opposite trend was observed. Moreover, Czechia has the highest geographical imbalance of nurses of all European countries (the comparison of Prague and the Central Bohemia region) [21].

A range of effective measures aimed at training sufficient numbers of nurses have been introduced in Nordic countries, which boast some of the highest numbers of nurses per capita in Europe and, moreover, with some of the youngest average age structures [13]. In Norway, for example, the government has been implementing action plans since 2016 aimed at improving the working conditions of nurses, expanding their competencies and introducing measures to motivate more students to study nursing [22]. In Finland, the expansion of the competencies of nurses even helps to address the shortage of doctors, primarily in remote areas where the capacities of doctors are most at risk [23].

In order to plan for the future capacity needs of nurses and develop effective policy strategies for stabilizing their numbers and their retention in the Czech healthcare system, it is necessary to form an understanding of their gender-age and geographical distributions, considering both ageing and movement within the system. Therefore, this article aims to provide a detailed demographic analysis of the nursing workforce in Czechia, focusing on age and geographical distribution. In addition, the paper provides a description of the capacities and structures of nurses by the type of healthcare provider (outpatient and inpatient care), patterns of entry into and exit from the healthcare system, and model-based projection of potential future capacity developments under certain assumptions. The findings are intended to inform evidence-based health workforce policy and planning.

## Data and methodology

This study was designed as a retrospective observational study combining trend analysis with a projection model. It applies demographic methods commonly used in projection analyses.

Due to the need for detailed data for targeted analysis purposes and the lack of data contained in publicly accessible sources at the time of the study, data obtained from the General Health Insurance Company of the Czech Republic (GHIC) [24] were used for the analysis of nurses in Czechia. This data was obtained on the basis of a cooperation agreement between the Faculty of Science of Charles University and the GHIC. The GHIC is the largest Czech health insurance company and has contractual agreements with almost all the country’s providers (almost 100%); hence, we had an almost complete source of data at our disposal. The data file contained individual anonymised data on non- medical healthcare workers, from 31 December 2012 to 30 September 2023, thus covering the most up-to-date data available.

### Definition of nurses and included variables

The dataset classifies non-medical healthcare workers into two main categories: ‘S‘^1^ and ‘SBM‘ 2. Category ‘S’ are defined as those with a minimum of a secondary school education with a leaving certificate obtained from a secondary medical school (medical assistant, practical nurse). A minimum of three years of higher or higher vocational medical school education is required for the general and paediatric nursing specialisations. Consequently, this analysis focuses exclusively on Category ‘S’, as it encompasses the qualified nursing workforce. Category ‘SBM’ workers were excluded from the analysis as they represent auxiliary staff without the required nursing qualifications.

The variables analysed included the worker’s age, gender, full-time equivalent (FTE), specialisation, type of care provider and, for the geographical analysis, data on the place of work at the NUTS 3 level. The healthcare system provides two main types of healthcare: outpatient and inpatient. Inpatient care is divided into acute care, follow-up care and health resorts and sanatoriums. The study also considered differences within individual specialisations (fields and areas of expertise). The specialisations selected for the analysis comprised those with the highest number of representatives or those that were otherwise considered important.

### Age and gender structure

The analysis directly addressed both the gender and age structures and the movement of nurses within the healthcare system. Basic descriptive statistical methods were employed to describe the structures, including graphical representations, e.g. population pyramids, which are used frequently in the field of demography. To highlight the replacement dynamics, the workforce was divided into the main three age categories: under 35, 35–59, and 60+. The 60 + category was specifically chosen to capture the immediate retirement dynamics in the Czech context. During the study period, the statutory retirement age for women ranged between 60 and 65 years, depending on the year of birth and the number of children raised. Consequently, the comparison differentiated between the ‘incoming’ generation (entries, < 35) and the ‘outgoing’ generation (exits, 60+) to emphasize the potential replacement deficit. The middle age group (35–59) represents the stable core of the workforce with relatively low fluctuation.

One of the specific calculations that reflects the age structure of nurses concerned the mean age of workers based on the application of weights, which corresponded to the full-time equivalent (FTE) capacity of workers. This calculation was chosen in order to capture the real mean age in terms of reflecting the “care provided”:

$$\overline{_tX^{s,p}}=\frac{\sum\left(_tx^{s,p}+0,5\right)\times{_t}u^{s,p}}{\sum_tu^{s,p}}$$

where $\overline{{{_{t}^{\:}X}^{s,p}}}$ denotes the mean age in year $\:t$, with specialisation $\:s$ or type of care $\:p$; $_tx^{s,p}$ is the completed age of the person in year $\:t$, with specialisation $\:s$ or type of care $\:p$; $_tu^{s,p}$ is the FTE capacity of the person in year $\:t$, with specialisation $\:s$ or type of care $\:p$.

### Probabilities of entry and exits

The movement of healthcare personnel was quantified using the probabilities of entering and exiting the system between ages $x$ and $x+1$, regardless of gender (due to the very low numbers of male nurses). Given the availability of unique detailed and accurate data, the article applied the cohort (generational) perspective, which is a useful approach to the estimation of healthcare worker departures with concern to tracking workers over time. The principle of calculating the probability of departure was adopted from a previous article by Havelková and Šídlo [25], in which the same indicator was calculated, but for a different group of healthcare workers, i.e. physicians:

$${}_t^cq_x^{x+1}=\frac{{}_t^cO_x^{x+1}}{{}_{31.12.t-1}\;^cP_x}$$

where ${}_t^cq_x^{x+1}$ is the probability of exits from the system during year $\:t$ between ages $x$and $x+1$, ${}_t^cO_x^{x+1}$ represents the number of nurses who exit the system in year $\:t$ (based on the tracking of the presence of individual workers on the list of nurses at both the end of year $\:t-1$ and the end of year $\:t$; if the worker is not listed in year $\:t$, they are considered to have exited the system) and $\:{}_{31.12.t-1}{}^{c}{P}_{x}$ is the number of nurses at the end of year $\:t-1$; $\:c$ is the generation (the year of birth) of the nurses.              

The probabilities were then smoothed applying the five-year moving average method. For the sake of simplicity, which was sufficient for the purposes of the analysis, a simple assumption was made for the probability of exit at older ages, namely a constant probability of exit at a level of 1 from the age of 85+.

The analysis also considered the entry of nurses into the system, for which the relative shares of entries were used for quantification purposes. The relative share of entering nurses was calculated as the average for 2019, 2020 and 2022 for the subsequent projection. These years were chosen since they represented the most recent years with complete data as of December 31. Data for 2021 were excluded due to specific administrative distortions related to the COVID-19 pandemic. In 2021, extraordinary financial bonuses were introduced for pandemic-related work, which resulted in a temporary artificial increase in the reported number of nurses, particularly among outpatient service providers (see Fig. 3) [5]. Since the years 2020 and 2022 were not affected by this specific administrative intervention, they were retained in the analysis.

### Projection model

The projection was subsequently compiled applying the above-mentioned probabilities of exit and the relative shares of entries into the healthcare system. The threshold for the projection was set at 2022, to which the average probability of exit and the relative share of entries from the values for 2019, 2020 and 2022 were applied for the reasons mentioned above. A calculation based on the cohort-component method was employed, in which the total number of entries was again calculated from the average of the entries for the three considered years.

$${}_{t+1}P_{x+1}={}_tP_x-\left({}_t^cq_x^{x+1}\times{}_tP_x\right)+\left(\frac{_tE_{x+1}}{\sum_tE_x}\times\overline{E}\right)$$

where ${}_t^cq_x^{x+1}$ is the probability of exits from the system during year $\:t$ between ages $x$and $x+1;{}_{t}^{\:}{P}_{x}$ is the number of persons in the system as of $\:31.\:12.$ in year $\:t$ and age $x$; $_tE_x$ is the number of persons entering the system during year $\:t$ and at age $x+1$ and $\:\stackrel{-}{E}$ is the selected constant (estimate) of the total annual number of persons entering the system; $\:c$ is the generation (year of birth) of the nurses.    

This calculation was used to make projections of the nursing populations in 2030 and 2035. In terms of the determination of the number of FTEs, it was sufficient to apply the average FTEs according to age. A constant value of 0.6 FTE was applied to nurses aged 85 and above due to the volatility of the data for the older age groups.

## Results

### Gender and age structures of the nurses

**Fig. 2:**
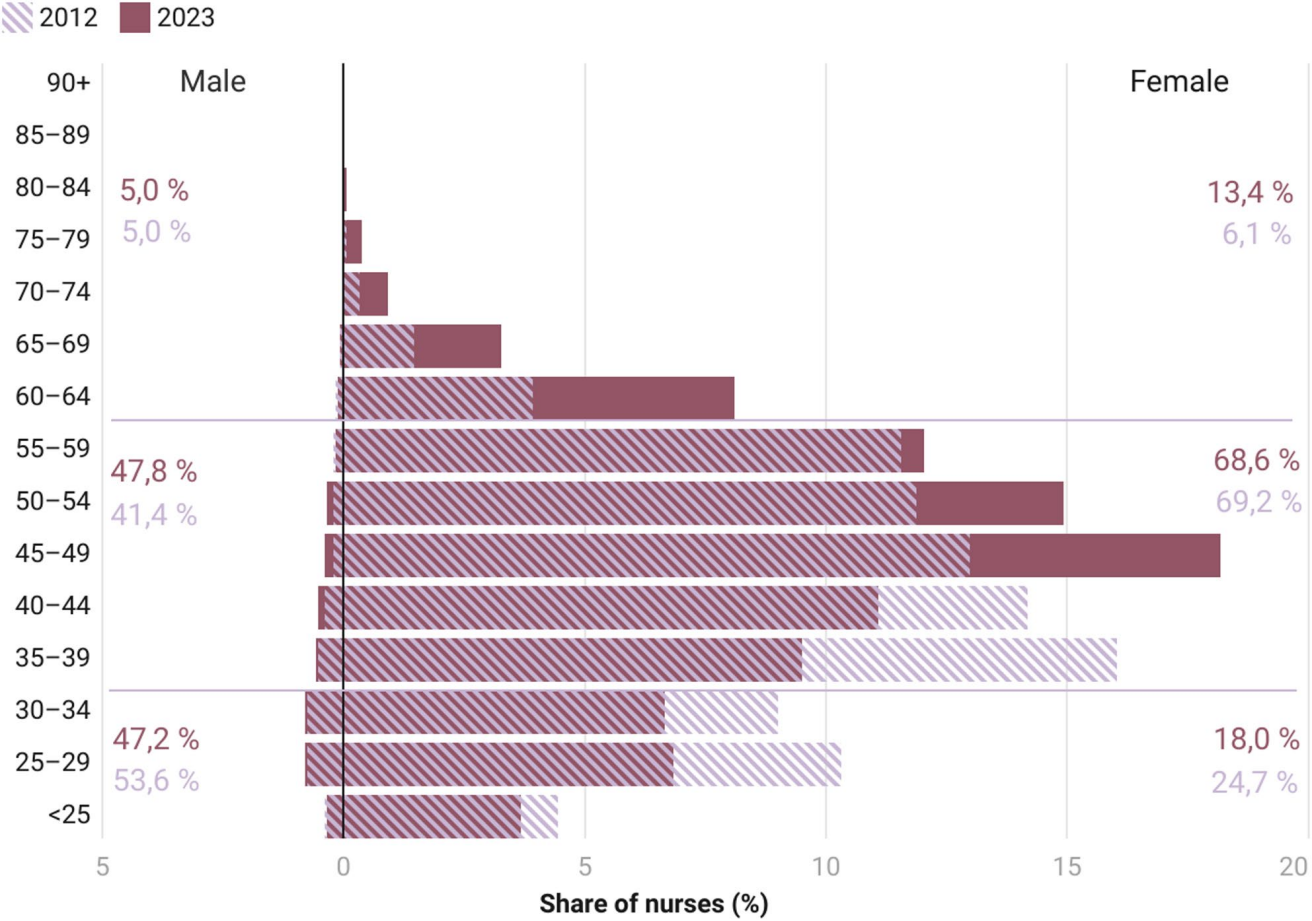
Age pyramid, structure of the nursing workforce based on Full-Time Equivalents (relative share), Czechia, 31.12.2012 and 30.9.2023. Note1: The horizontal lines divide the population into three main age cohorts: <35, 35–59, and 60+. The floating percentage values represent the aggregated share of each specific age cohort within the total workforce for 2012 (lighter) and 2023 (darker). (Total FTE capacity of nurses: N_2012= [87 507,6]; N_2023= [102 635,4]). Note2: When comparing the age structures from 2012 and 2023, it is necessary to take into account that they may have been slightly affected by legislative changes, primarily in the areas of other care providers and health resorts. Source: [24]; author’s own calculations

The gender structure of nurses has historically been very uneven. In 2023, women accounted for almost 96% of nurses, with the highest proportion in the 45–49 age group (Fig. 2). The male component of this workforce, in contrast, is more highly represented by younger age categories. The comparison of the initial year under review, 2012, and the last year, 2023, revealed a significant shift in the age structure, i.e. increases in the proportions of all the age categories from 45 years and above and, conversely, a significant decrease in the representation of younger age categories (Fig. 2).

Just as the population generally is ageing for two reasons, i.e. higher life expectancy accompanied by a decrease in the number of births [26, 27], the representation of nurses in older age categories is increasing in tandem with decreasing numbers of new entrants to the profession. Whereas this trend is most visible for women, the slight strengthening of this trend is also evident for men.

**Fig. 3:**
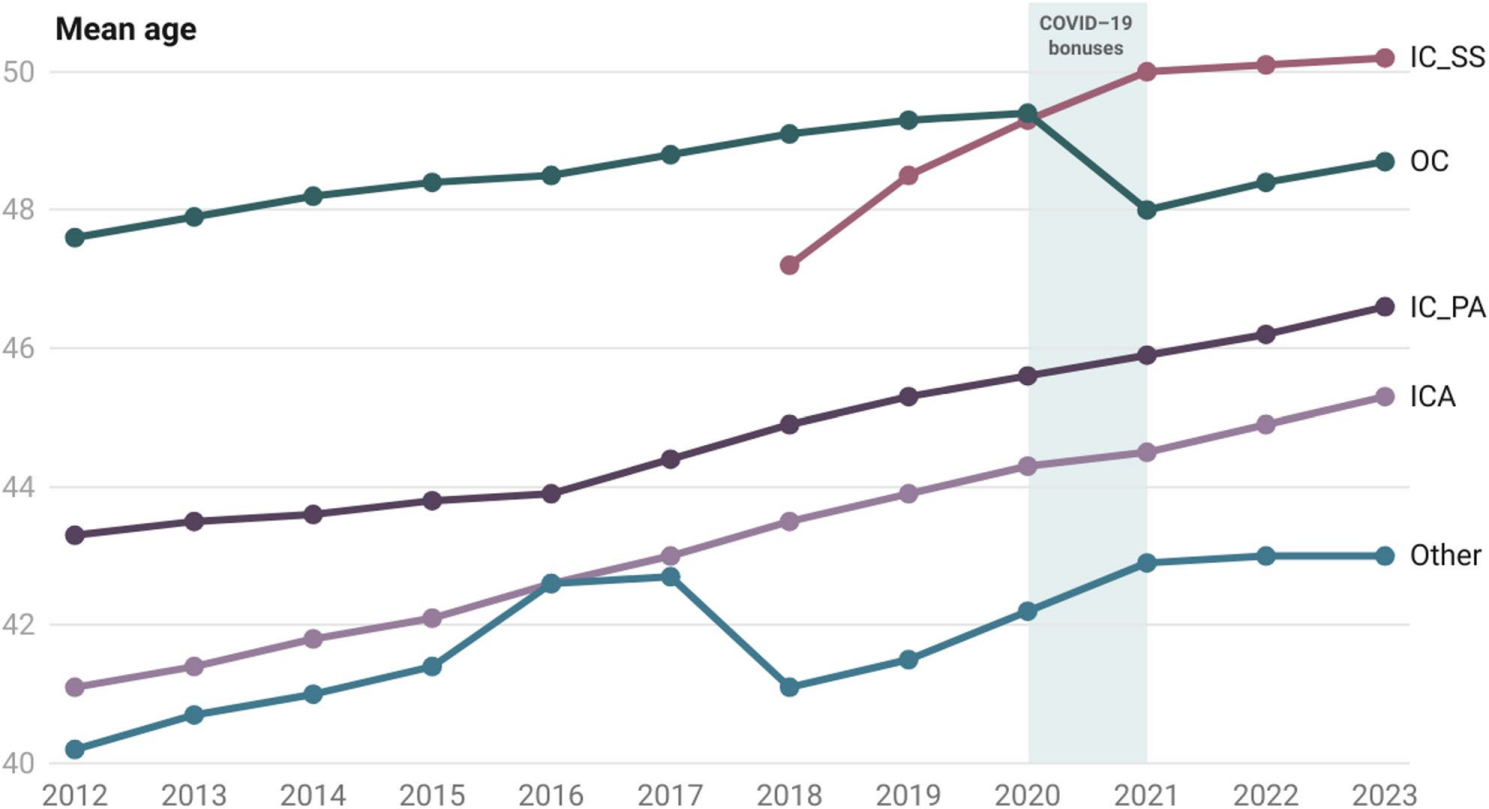
Mean age of nurses by type of healthcare provider, Czechia, 2012–2023 (as of 31.12. of the given year*). Note: * 2023 as of 30. 9. Note: OC = Providers of outpatient care; IC_A = Providers of acute inpatient care; IC_PA = Providers of post-acute inpatient care; IC_SS = Providers of inpatient services at health resorts and sanatoriums; Other = Other healthcare service providers. Source: [24]; author’s own calculations

A closer look at the individual categories by the type of healthcare provider indicates, with certain exceptions, increases in the mean age weighted by capacity for all the categories in recent years (Fig. 3). The exceptions are nurses who work for outpatient care providers and, in some years, those who work in other types of care, concerning whom significant changes occurred in terms of the number of full-time positions; thus, the fluctuations in the mean age were most likely caused by legislative changes. With respect to outpatient care, a significant decrease in the mean age, i.e. by almost 1.5 years (Fig. 3), was evident between 2020 and 2021. Given the timing of this significant decline during the COVID-19 pandemic, it can be assumed that the decrease was related to this event. We attributed the decline to an increase in the number of “approved” and subsequently registered positions that were not previously officially recorded, aimed at obtaining financial support during the COVID-19 pandemic [5]. Registration may have led to the provision of more accurate data than previously available and, thus, resulted in the lower mean age. The impacts of changes to the data and the afore-mentioned registration are evident from the total numbers of physical (headcounts) and full-time equivalent capacities, with an increase of 8,500 FTE from 2020 to 2021, 90% of which were in outpatient care, specifically nurses in the dentistry segment.

The highest mean age was recorded for nurses who work in sanatoriums and health resorts, concerning whom the mean age stood at more than 50 years in 2023. The mean age of nurses in outpatient care is almost 49 years. Nurses in inpatient follow-up care and inpatient acute care were observed to be younger on average.

**Fig. 4:**
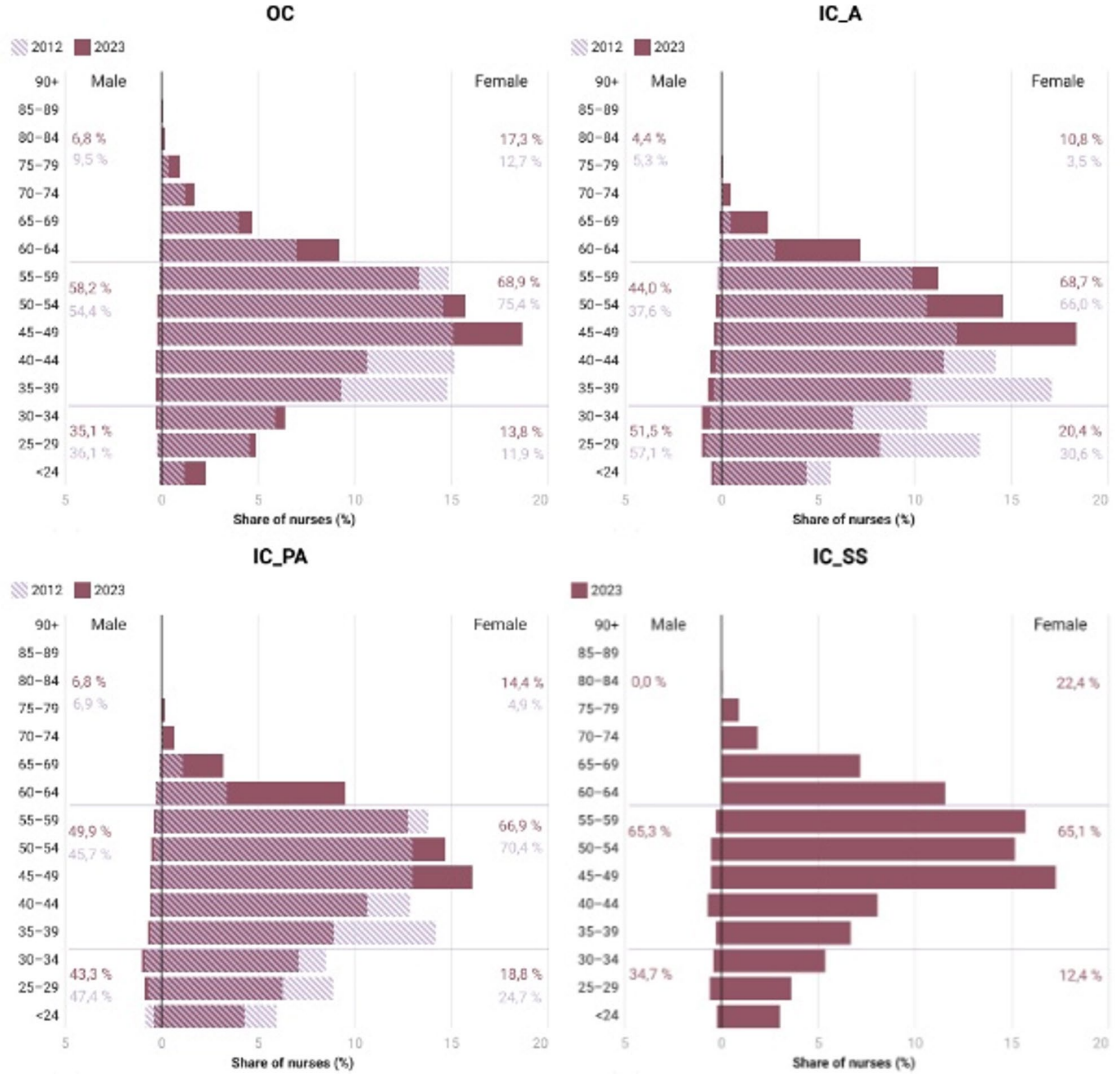
Age pyramids of nurses by selected types of care providers, Czechia, 31.12.2012 and 30.9.2023. Note: The contractual registration of sanatorium and health resort personnel commenced in 2018; therefore, the age structure in 2023 only is shown. Note: see Fig. 3. Source: [24]; author’s own calculations

A closer look at the age pyramids for the various types of care providers reveals two reasons for the ageing of the nursing population, both at the bottom of the pyramid, i.e. the declining proportions of younger age categories and, at the top, the increasing proportions of older categories (Fig. 4). This is particularly evident for acute inpatient and follow-up care. Since data on sanatoriums and health resorts is only available from 2018 onwards, it is not possible to compare this sector for the whole of the period 2012–2023; nevertheless, the age pyramid is interesting due to the significant difference between the proportions of younger and older age categories in 2023. The proportion of women aged 60 and over more than tripled in the acute inpatient care providers during the period under review. Moreover, an almost three-fold increase was evident for this age category in the follow-up inpatient care providers. In contrast, in 2023, there were almost 1.5 times fewer female nurses under the age of 35 in both these care providers. No decline in the younger age categories was evident for women in the outpatient care providers; however, an increase of almost 5 percentage points was observed for older age categories. The age distribution of male nurses remained relatively similar for all the types of care providers. It is important to bear in mind here the refinement of the data in 2021, primarily with concern to outpatient care, which may have influenced the comparison.

However, the detailed study of the structures of and potential capacity planning for nurses requires the consideration of the various specialisations and fields. Those specialisations and fields that are most numerous or otherwise significant were selected for analysis purposes.

**Fig. 5:**
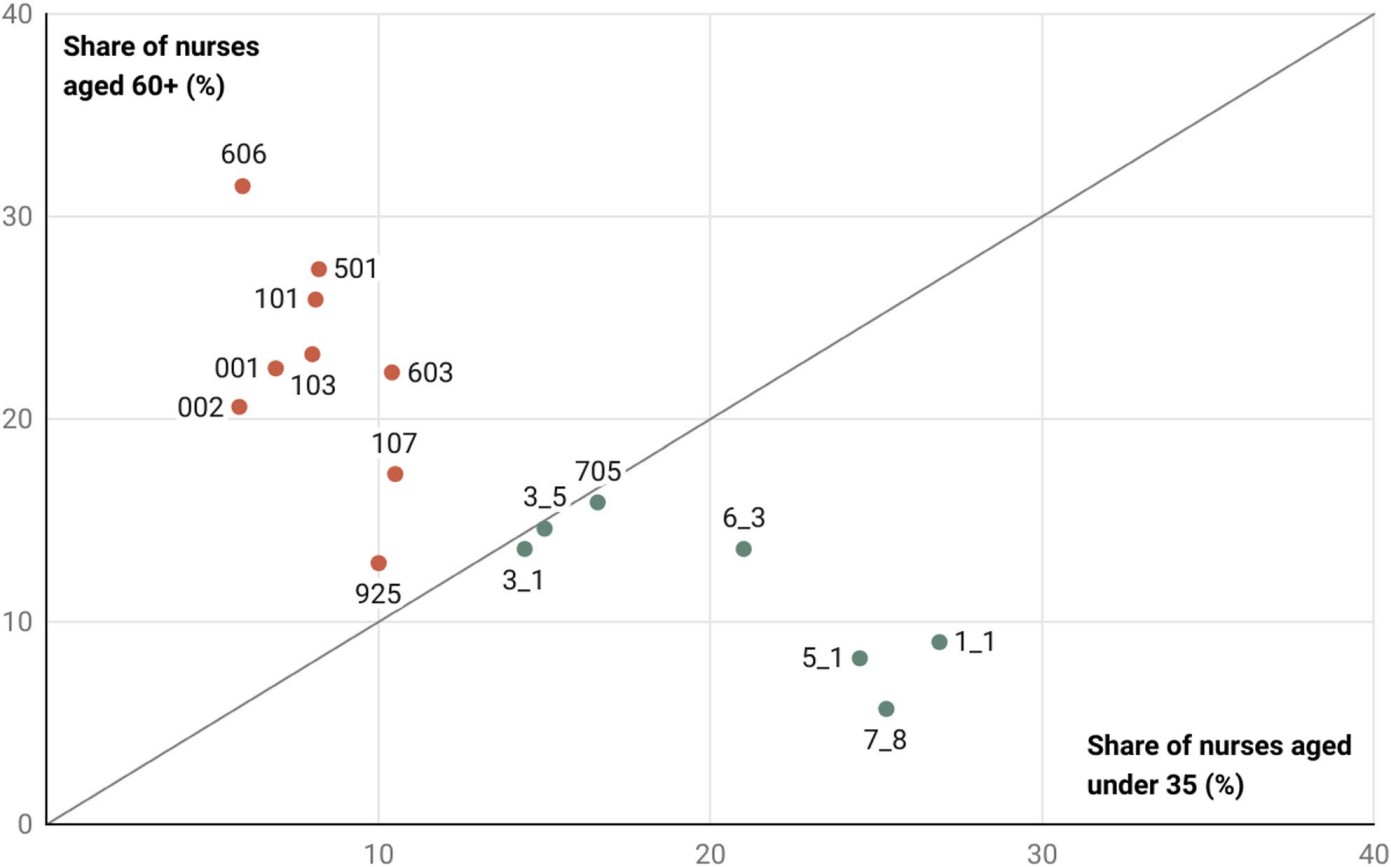
Share of nurses in the 60+ and under 35 age groups, selected specialisations and acute care fields, Czechia, 2023. Notes: Outpatient specialties: 001 – General practice, 002 – Paediatric general practice, 101 – Internal medicine, 103 – Diabetology, 107 – Cardiology, 501 – Surgery, 603 – Obstetrics and gynaecology, 606 – Orthopaedics, 705 – Ophthalmology, 925 – Home health care nurses. Inpatient care fields: 1_1 – Internal medicine, 3_1 – Paediatrics, 3_5 – Psychiatry, 5_1 – Surgery, 6_3 – Obstetrics and gynaecology, 7_8 – Anaesthesiology and resuscitation. Source: [24]; author’s own calculations

The identification of potential future problems requires the comparison of the older and younger age groups, i.e. whether there is sufficient younger personnel capacity available to replace older workers who plan to retire. Thus, the under 35 and 60+ age groups were compared (Fig. 5). Of the specialisations investigated, orthopaedics (606) proved to be particularly potentially under threat of capacity shortages. In general, all the fields with higher proportions of older than younger workers (marked in red in Fig. 5) were considered to be at risk. In contrast, a number of fields with relatively younger nurses and, thus, potentially lower risks of shortages, were identified, i.e. internal medicine (1_1), anaesthesiology and resuscitation (7_8) and surgery (5_1).

The geographical perspective is also an important factor. Since the geographical distribution and age structures of nurses is uneven across the various regions of Czechia, an analysis was subsequently performed of nurses at the NUTS3 level (Table 1). The differences are complex and could be described at length; however, for the purposes of this article it is sufficient to provide a summary of the basic results, which pointed to problems primarily in the Karlovy Vary region (CZ041) and in Prague (CZ010). In 2022, the Karlovy Vary region exhibited one of the highest nurses mean ages in the outpatient care provider (49.6 years), general practice (52.8 years) and acute paediatrics inpatient care categories (50.4 years). Prague (the capital of Czechia) is facing a particular problem with a significant increase in the mean age of nurses in the inpatient care category. Specifically, with concern to acute inpatient care, an increase was observed in the mean age of nurses by 4.5 years between 2012 and 2022. Prague also exhibited one of the highest mean ages of nurses working in general practice (52.6 years) and follow-up inpatient care (48.6 years) in 2022.

**Table 1:** Characteristics of the age composition of nurses and for individual types of care providers, Czechia, NUTS 3 regions, 2022

NUTS 3 region	OC (Providers of outpatient care)			IC_A (Providers of acute inpatient care)			IC_PA (Providers of post-acute inpatient care)		
	Share of nurses aged		Mean age	Share of nurses aged		Mean age	Share of nurses aged		Mean age
	–35 y. (%)	60+ y. (%)		–35 y. (%)	60+ y. (%)		–35 y. (%)	60+ y. (%)	
CZ010	17.8	18.5	48.3	22.4	12.2	45.6	18.0	18.4	48.6
CZ020	11.1	18.0	49.5	19.6	11.4	45.9	19.2	13.3	46.9
CZ031	11.8	14.9	48.6	25.0	9.9	44.3	21.9	12.2	45.9
CZ032	14.4	17.9	49.0	23.5	9.8	44.7	17.1	14.4	47.4
CZ041	13.8	19.4	49.6	24.9	12.3	45.0	33.2	14.2	42.8
CZ042	12.8	18.0	49.4	18.0	11.0	46.6	16.5	16.1	47.8
CZ051	12.6	18.5	49.6	24.8	9.2	44.3	19.7	13.4	46.6
CZ052	12.0	17.2	49.1	24.0	8.3	44.1	17.9	12.9	46.9
CZ053	18.6	12.6	46.3	27.1	7.4	42.8	22.3	11.3	45.4
CZ063	15.8	14.3	47.4	19.5	7.7	44.9	21.7	7.6	44.5
CZ064	16.7	16.3	47.9	24.5	8.5	44.1	20.6	13.8	46.1
CZ071	13.6	15.1	48.1	18.7	8.1	45.3	22.5	11.0	45.6
CZ072	12.8	15.1	48.2	28.5	7.9	42.9	23.7	8.2	44.4
CZ080	15.4	13.4	47.5	22.7	8.8	44.7	22.4	9.6	45.3

CZ010 – Prague, CZ020 – Central Bohemian Region, CZ031 – South Bohemian Region, CZ032 – Plzeň Region, CZ041 – Karlovy Vary Region, CZ042 – Ústí nad Labem Region, CZ051 – Liberec Region, CZ052 – Hradec Králové Region, CZ053 – Pardubice Region, CZ063 – Vysočina Region, CZ064 – South Moravian Region, CZ071 – Olomouc Region, CZ072 – Zlín Region, CZ080 – Moravian-Silesian Region

Source: [24]; author’s own calculations

### Movement of nurses in the healthcare system

The movement of nurses in the healthcare system was monitored aimed at forming a deeper understanding of changes in the age structure and, subsequently, the construction of projections. The probabilities of exit and the relative distribution of entrants were monitored (by age group in both cases).

**Fig. 6:**
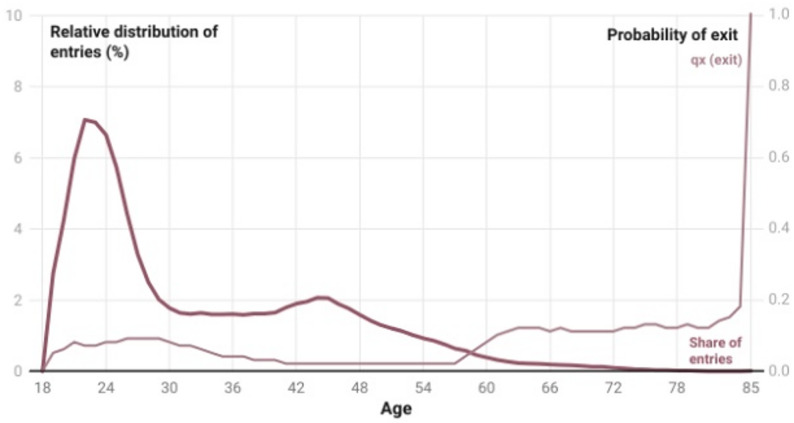
Share of nurses that entered the healthcare system and the probability of exit by age, Czechia, average for 2019, 2020 and 2022 Source: [24]; author’s own calculations

**Fig. 7:**
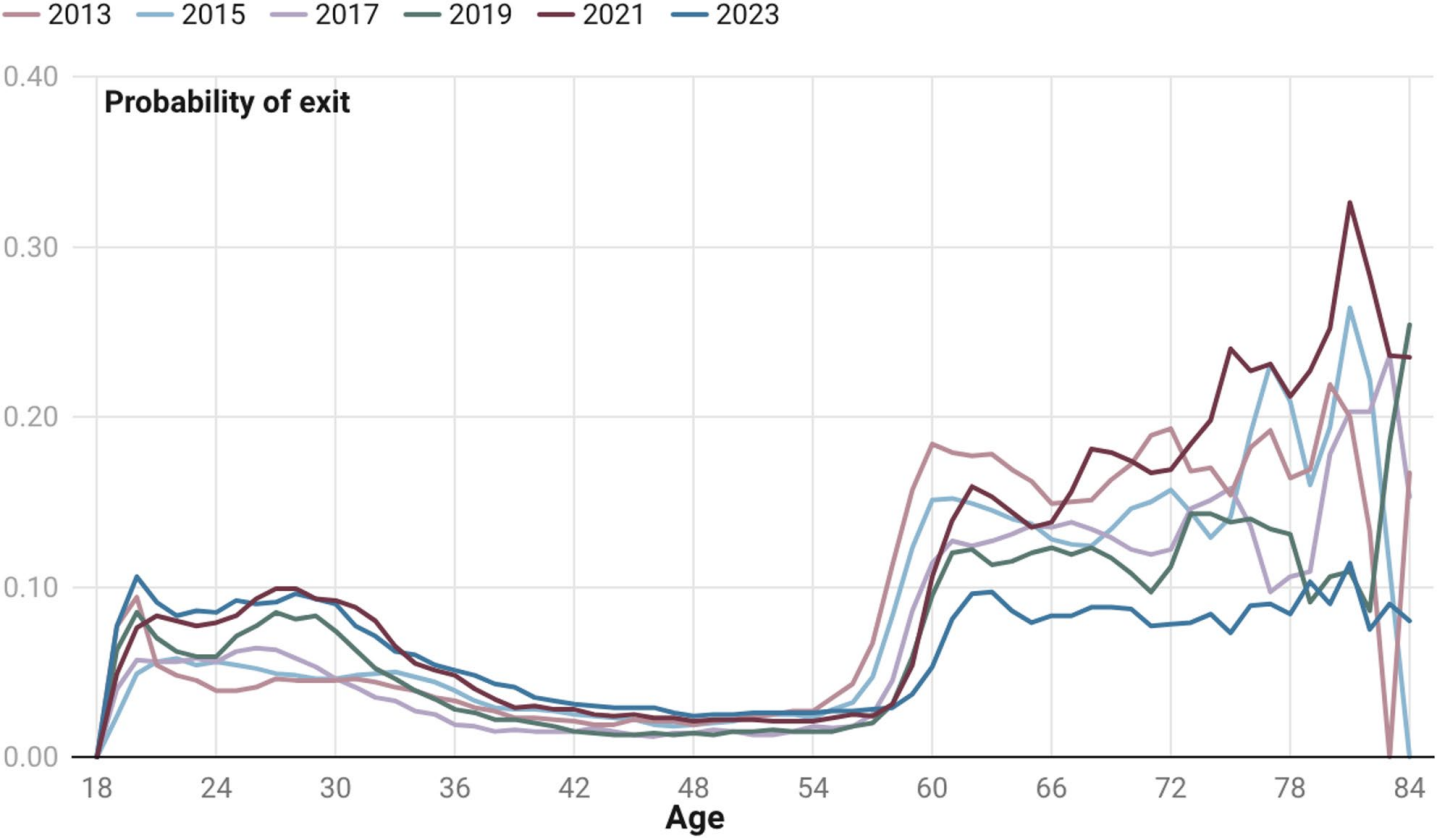
Adjusted probabilities of nurses exiting the profession, Czechia, selected years 2012–2023 Source: [24]; author’s own calculations

The entry analysis indicated that the highest number of entries occurred immediately following graduation at around the age of 22, followed by a decline up to around the age of 42, when the probability once more begins to increase slightly up to the age of 46, from which point a decline is evident (Fig. 6). The average probability of exit increases immediately after graduation, most probably due to maternity and parental leave. Subsequently, the probability remains very low between the ages of 38 and 57, followed by an increase upon reaching retirement age (Fig. 6). From the age of 85+, in light of the highly volatile values observed, the exit from the system was assumed of all workers either due to death or the personal decision to terminate their employment. The development of the probability of exit over the period 2012–2023 was of particular interest (Fig. 7). The curves illustrate the development of the probability of exiting the system during two key periods immediately after the completion of study, when the probability gradually increased over time, and later around retirement age, when the rate of increase slows down and the exits shift to older ages.

The projection of future developments was created based on the average probabilities and the relative distribution of entries. The projections of the nursing population up to 2030 and 2035 were based on the data as of 31 December 2022. A constant number of entrants was assumed based on the average for recent years, i.e. 4,775 nurses per year. The average FTEs by age were used to estimate the FTE capacities of the nurses. 

**Fig. 8:**
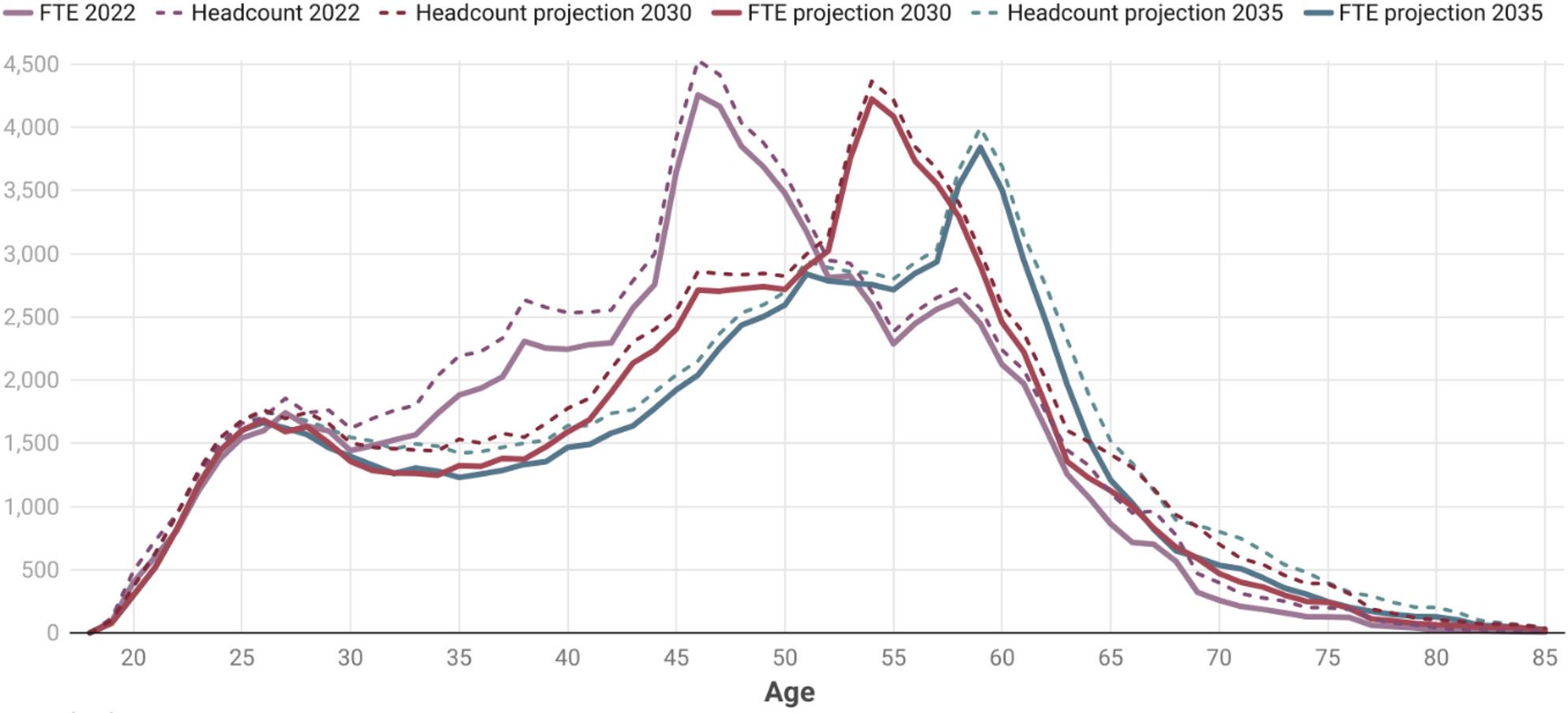
Age distribution of physical numbers (headcounts) and full-time equivalent capacities of nurses, Czechia, 2022, projections for 2030 and 2035 Source: [24]; author’s own calculations

The projections pointed to declines with respect to both the projected years (2030 and 2035) compared to the baseline. If the conditions assumed for the projections are accurate, declines will occur of approximately 3.5% (4,000 nurses) and more than 8% (8,000) by 2030 and 2035, respectively (Table 2). This would result in significant shifts in the age structures towards the ageing of the nursing population (Fig. 8), i.e. an increase in those aged 60+ of 4 percentage points by 2030 and of 9.1 percentage points by 2035, accompanied by a decrease in the younger age category (–35 years) of 0.9 percentage points by 2030 and of up to 2.6 percentage points by 2035 (Table 2).

**Table 2:** Assumptions and results of the projection for nurses, Czechia, 2022 and projections for 2030 and 2035 (as of 31.12. of the given year)

Indicator	2022	Projection 2030	Projection 2035	Difference 2030 − 2022	Difference 2035 − 2022
Annual number of entrants	4,890	4,775	4,775	-115	-115
Annual number of exits	5,952	5,412	5,780	-540	-172
Annual balance	-1,062	-637	-1,005	425	57
Headcounts	112,521	108,252	104,021	-4,269	-8,500
FTE	102,346	98,725	94,179	-3,621	-8,167
Share of FTEs aged under 35 years	19.8	18.9	17.2	0.9*	2.6*
Share of FTEs aged 60 years and over	12.3	16.3	21.4	4.0*	9.1*

*percentage points

Source: [24]; author’s own calculations

## Discussion

The analysis indicated the ageing trend among nurses across all types of healthcare providers in Czechia in recent years. Declines are evident in terms of the number of nurses in the younger age categories and increases in the proportions of older nurses. The unfavourable age structure of nurses is influenced by both the declining number of nursing students [28] and frequent exits prior to retirement age. Similar trends have been observed in other countries and have often been linked to demanding working conditions [9].

Interestingly, regarding regional disparities, the highest mean age of nurses in inpatient care was observed in the capital city, Prague, which also exhibited the highest increase in the mean age during the studied period. This finding is contrary to that of most other similar studies, which revealed that the mean age of nurses is higher in rural than in urban areas [29]. One explanation for this specific trend could be the higher cost of living in the metropolitan area, necessitating that nurses remain in the workforce longer, often extending their careers beyond the statutory retirement age. Conversely, the high mean age observed in outpatient care in the Karlovy Vary region may be attributed to the specific socio-economic conditions of border regions. These areas are generally less attractive to younger generations. Furthermore, the proximity of these regions to Western labour markets facilitates the cross-border migration of younger nurses seeking better financial conditions, leaving the local system reliant on an ageing workforce.

Compared with primary care physicians, whose exits increase gradually with age and often occur beyond the statutory retirement age [25], nurses in Czechia tend to leave the workforce primarily around retirement age. The legal retirement age in Czechia depends on the year of birth and, for those born before 1965, also on the gender and number of children raised. The retirement age is set at 65 for those born after 1965 regardless of gender or family size [30]. The shift of exits from the system of nurses to a higher age group between 2013 and 2023 is therefore partly due to the gradual increase in the legal retirement age.

A concerning finding is the increasing probability of exiting the healthcare sector at the beginning of nurses’ career. This trend reflects dissatisfaction with working conditions, challenges in achieving work-life balance, or insufficient support during the transition to practice [31, 32]. The experiences of newly-graduated nurses have been summarized in terms of three main themes: a feeling of a lack of competence, feeling of emotional distress, and the need for support [33]. Such challenges underline the importance of retention interventions targeting newly graduated nurses.

General reasons for healthcare workers deciding to exit the profession are also complex and influenced by a combination of workload, financial and personal factors (e.g. health), social recognition and global trends, including migration [34]. Nurses face higher levels of physical and emotional stress, lower salaries and lower professional recognition than doctors, which may contribute to burnout [32]. Studies have also pointed out that the dissatisfaction of nurses due to staff shortages and the associated poor working conditions may lead to further exits [35, 36]. These pressures were intensified by the COVID-19 pandemic [37]. Although a temporary increase occurred in the number of active nurses in Czechia during the pandemic, a significant decline in new entrants to the profession was observed in subsequent years. Understanding these factors is essential in terms of designing targeted strategies, e.g. the introduction of burnout mitigation programmes, including oversight similar to that applied in social services.

Our projections show that if the current number of new entrants were to be maintained, capacity would decline by more than 8% by 2035. According to experts, the current capacity is already insufficient [5, 12]; thus, in combination with the increase in the demand for healthcare due to the ageing population in Czechia, the predicted decline will create serious capacity-related problems. Forecasts indicate that the number of nurses will need to increase by approximately 1.7–2.6 times by 2050 [38]. These predictions underscore the need to implement measures that will reduce early exits and, at the same time, encourage the recruitment of new students for nursing training programmes. In 2025, the Czech government responded to this situation by financially supporting a programme that is intended to increase the number of nursing students by 20% over the next 12 years. This increase reflects both the generational change of these workers and the increased demand caused by the ageing of the Czech population [18, 38]. However, without simultaneous measures to reduce attrition and improve retention, the long-term impact of this policy will likely be limited.

Recent studies point to the growing trend of attrition among nursing students [39]. A Czech study reported that 12.2% of nursing students in Czechia were unsure as to whether they would pursue a nursing career, and 1.8% stated that they did not intend to continue in the profession [17]. This highlights the need for supportive adaptation programmes to facilitate the transition from graduation to practice – the first year of work experience is often decisive [40, 41]. However, the fact that, according to national register data, more than 80–90% of nursing graduates work in the public health insurance system [38], indicates that this problem is not yet acute in Czechia.

Furthermore, gender inequality has been reported as being a significant factor with respect to the nursing profession in Czechia. The lack of attention being devoted to gender issues – e.g. support for childcare, care of elderly relatives and the availability of flexible working arrangements –is potentially exerting a negative impact on the quality of healthcare services [42, 43]. Policies aimed at supporting work–family reconciliation, such as flexible scheduling and part-time opportunities, could therefore contribute significantly to workforce sustainability.

Finally, but no less important, inequalities across regions and types of healthcare providers warrant closer policy attention. Thus, further research should build upon the detailed regional and profession-specific analyses complemented with education data in order to provide more accurate data for the determination of targeted intervention measures. Additionally, more granular analyses of migration patterns and the impact of foreign-born nurses on the Czech labour market are needed. Qualitative analyses on drivers of nurses leaving the profession could also provide deeper insights.

### Limitations

This study has several limitations. The projection does not incorporate data on the number of nursing students or their graduation success rates, as these data are not available at the individual level. Similarly, migration trends were not modelled due to their high complexity and low predictability. Although Czechia has not yet experienced drastic migration fluxes compared to some other nations, this factor warrants close monitoring. Therefore, the study represents a simplified yet sufficiently robust model for illustrating overall workforce dynamics. The projection should be interpreted as a “what-if” scenario, showing how personnel capacities in the healthcare sector might evolve under the assumption that current entry and exit patterns remain constant, rather than as a precise forecast of future values. Moreover, although the dataset from the General Health Insurance Company covers almost all healthcare providers in Czechia, it may still omit a small fraction of nurses working outside the public insurance system. Despite these limitations, the findings provide valuable evidence for understanding long-term workforce dynamics and informing strategic planning.

## Conclusion

The results revealed that the ageing of nurses in Czechia represents a serious structural challenge for the national healthcare system. The age structure is shifting towards older age categories and the proportion of younger age groups is steadily declining. If current entry and exit trends continue, projections for 2030 and 2035 predict declines in both the full-time and physical (headcounts) capacities of 3.5% to 8%. These findings highlight the need for systemic and long-term workforce strategies focused on improving working conditions, strengthening early-career support, and increasing the attractiveness of nursing education programmes. Particular attention should be devoted to those regions with the highest rates of ageing, as well as to those specialisations with low proportions of younger workers.

Furthermore, it is important to note that the decisions of nurses to remain in or to leave the system are influenced by many factors, including age, working conditions, professional recognition, and opportunities to balance work and personal life. The timing and intensity of entries and exits change over time, which should be reflected in dynamic workforce planning models.

It is hoped that the findings of this study may be valuable for other countries that are facing similar challenges related to the ageing of the healthcare workforce, particularly in the field of nursing. The main problem issues — low proportions of young people, higher rates of departure in middle age, uneven regional distribution — are similar for most European countries. By applying demographic methods to workforce planning, this research contributes an evidence base for health policy decision-making in ageing healthcare systems.

## Data Availability

The data that supports the findings of this study is available from the General Health Insurance Company (GHIC); however, restrictions apply to the availability of this data, which was used for the purposes of this study under contract, and is, therefore, not publicly available. However, the data is available from the authors upon reasonable request and with the consent of the GHIC.

## Footnotes

1 S1 – Non-medical healthcare professional qualified to practice healthcare under professional supervision or direct guidance (ZPOD). S2 – Non-medical healthcare professional qualified to practice healthcare without professional supervision (ZPBD). S3 – Non-medical healthcare professional qualified to practice healthcare without professional supervision with specialized qualifications (ZPBD with relevant specialization) or special professional qualifications (ZPZS). S4 – Non-medical healthcare professional with university education, specialized qualification, or special professional qualification. (From 2022, only addiction specialists; others will be gradually transferred to S3).

2 SBM – Non-medical healthcare professional qualified to practice healthcare under professional supervision or direct guidance, without a secondary school-leaving certificate (auxiliary healthcare staff).

## Abbreviations

FTE: Full-time equivalent
GHIC: General Health Insurance Company
IC_A: Providers of acute inpatient care
IC_PA: Providers of post-acute inpatient care
IC_SS: Providers of inpatient services at health resorts and sanatoriums
OC: Providers of outpatient care

## References

[1] Bloom DE, Canning D, Lubet A. Global population aging: facts, challenges, solutions & perspectives. Daedalus. 2015;144(2):80–92. 10.1162/DAED_a_00332.

[2] Lutz W, Sanderson W, Scherboff S. The coming acceleration of global population ageing. Nature. 2008;451:716–9. 10.1038/nature06516. 18204438 10.1038/nature06516

[3] Prince M, Wu F, Guo Y, et al. The burden of disease in older people and implications for health policy and practice. Lancet. 2014;385(9967):549–62. 10.1016/S0140-6736(14)61347-7. 25468153 10.1016/S0140-6736(14)61347-7

[4] World Health Organization. State of the world’s nursing 2020: investing in education, jobs and leadership. Geneva: World Health Organization; 2020.

[5] Válek V, Dušek L, editors. Strategic analyses of healthcare sector needs: A concept based on available data [Strategické analýzy potřeb resortu zdravotnictví: Koncepce podložená dostupnými daty]. Praha: Ministerstvo zdravotnictví ČR; 2024. https://www.nzip.cz/koncepce2025. Czech.

[6] Harrington L, Heidkamp M. The Aging Workforce: Challenges for the Health Care Industry Workforce. New Brunswick, NJ: John J. Heldrich Center for Workforce Development, Rutgers University; 2013.

[7] Liu JX, Goryakin Y, Maeda A, et al. Global health workforce labor market projections for 2030. Hum Resour Health. 2017;15(11). 10.1186/s12960-017-0187-2. 10.1186/s12960-017-0187-2 PMC5291995 28159017

[8] Drennan VM, Ross F. Global nurse shortages—the facts, the impact and action for change. Br Med Bull. 2019;130(1):25–37. 10.1093/bmb/ldz014. 31086957 10.1093/bmb/ldz014

[9] Tamata AT, Mohammadnezhad M. A systematic review study on the factors affecting shortage of nursing workforce in the hospitals. Nurs Open. 2023;10(3):1247–57. 36303066 10.1002/nop2.1434 PMC9912424

[10] OECD. What do we know about young people’s interest in health careers? Paris: OECD Publishing; 2025. 10.1787/002b3a39-en.

[11] OECD. OECD Health at a Glance 2024: OECD Indicators. Paris: OECD Publishing; 2024. 10.1787/b3704e14-en.

[12] Koubová M. Increased capacity of universities for nurses and other non-physician healthcare professionals could be a reality within a year, says Chief Nurse of the Czech Republic, Alice Strnadová. [Navýšení kapacit vysokých škol pro sestry a další nelékařské zdravotníky by mohlo být už za rok, říká hlavní sestra ČR Alice Strnadová]. *Zdravotnický deník*. 2023. https://www.zdravotnickydenik.cz/2023/05/navyseni-kapacit-vysokych-skol-pro-sestry-a-dalsi-nelekarske-zdravotniky-by-mohlo-byt-uz-za-rok-rika-hlavni-sestra-cr-alice-strnadova/. Accessed 3 August 2025. Czech.

[13] World Health Organization. National Health Workforce Accounts Data Portal. WHO. 2022. Dostupné z: https://apps.who.int/nhwaportal/Home/Index. Accessed 5. 9. 2023.

[14] World Health Organization. State of the World’s Nursing 2020: Investing in Education, Jobs and Leadership. Geneva: World Health Organization; 2020. Licence: CC BY-NC-SA 3.0 IGO.

[15] Fischer J, Mazouch P, Vltavská K. A Methodology for estimating the number of students and graduates needed for general nurse education programmes [Metodika odhadu potřebného počtu studentů a absolventů studijních programů zaměřených na vzdělávání všeobecných sester]. VŠE; 2021. https://mzd.gov.cz/wp-content/uploads/2022/11/Metodika-odhadu-potrebneho-poctu-studentu-a-absolventu-studijnich-programu-zamerenych-na-vzdelavani-vseobecnych-sester.pdf. Czech.

[16] Ministerstvo zdravotnictví České republiky. Nursing concepts [Koncepce ošetřovatelství]. Praha: Ministerstvo zdravotnictví ČR; 2021. https://mzd.gov.cz/koncepce-osetrovatelstvi/.Czech.

[17] Večerková D, Gilchrist A, Riad A, Pokorná A. Examining work ability in nursing students: the role of job demand, control, and social support. Nurse Educ Pract. 2025;86(104404). 10.1016/j.nepr.2025.104404. 10.1016/j.nepr.2025.104404 40414023

[18] Kut Citores F. The government has approved a program worth 13 billion to educate nurses and other non-physician healthcare professionals [Vláda schválila program za 13 miliard na vzdělávání sester a dalších nelékařů]. *Medical Tribune*. 16. 7. 2025. https://www.tribune.cz/vsechny-clanky/vlada-schvalila-program-za-13-miliard-na-vzdelavani-sester-a-dalsich-nelekaru/. Accessed 16 April 2025. Czech.

[19] Kroezen M, Hoegaerden MV, Batenburg R. The joint action on health workforce planning and forecasting: results of a European programme to improve health workforce policies. Health Policy. 2018;122(2):87–93. 10.1016/j.healthpol.2017.12.002. 29241846 10.1016/j.healthpol.2017.12.002

[20] Schofield DJ, Page SL, Lyle DM, Walker TJ. Ageing of the baby boomer generation: how demographic change will impact on city and rural GP and nursing workforce. Rural Remote Health. 2006;6(4):1–9. 17061915

[21] Winkelmann J, Muench U, Maier CB. Time trends in the regional distribution of physicians, nurses and midwives in Europe. BMC Health Serv Res. 2020;20(937). 10.1186/s12913-020-05760-y. 10.1186/s12913-020-05760-y PMC7549210 33046077

[22] Organisation for Economic Co-operation and Development. Health at a Glance 2021: OECD Indicators. Paris: OECD Publishing. 2021. 10.1787/ae3016b9-en. Citováno 7. 5. 2023.

[23] Rafferty AM et al. Strengthening health systems through nursing: Evidence from 14 European countries. Copenhagen (Denmark): European Observatory on Health Systems and Policies; 2019. https://www.ncbi.nlm.nih.gov/books/NBK545724/31465161

[24] GHIC CR. Individual anonymized data provided for scientific purposes under an agreement between General Health Insurance Company of the Czech Republic and Charles University, Faculty of Science. General Health Insurance Company of the Czech Republic; 2023.

[25] Havelková T, Šídlo L. Working life expectancy of physicians: the case of primary care physicians in Czechia. Hum Resour Health. 2025;23(1):9. 10.1186/s12960-025-00978-5. 39939999 10.1186/s12960-025-00978-5 PMC11823157

[26] Poston DL, Bouvier LF. Population and society: an introduction to demography. Cambridge: Cambridge University Press; 2010.

[27] FERNANDES F, TURRA CássioM, RIOS-NETO, Eduardo LG. World population aging as a function of period demographic conditions. Demographic Res. 2023;48:353–72.

[28] Eurostat. Graduates in nursing, midwifery and care. Statistics Explained. 2022. https://ec.europa.eu/eurostat/statistics-explained/index.php?oldid=664. Accessed 16 July 2025.

[29] Smith JG, Plover CM, McChesney MC, Lake ET. Isolated, small, and large hospitals have fewer nursing resources than urban hospitals: Implications for rural health policy. Public Health Nurs. 2019;36(4):469–77. 10.1111/phn.12612. 30957926 10.1111/phn.12612 PMC6635079

[30] Czech Republic. Act No. 155/1995 Coll. Pension Insurance Act [Zákon č. 155/1995 Sb. Zákon o důchodovém pojištění]. https://www.zakonyprolidi.cz/cs/1995-155. Czech.

[31] Aamir A, et al. Work-life balance, job satisfaction and nurses retention: moderating role of work volition. Int J Bus Excell. 2016;10(4):488–501.

[32] Karaferis D, et al. Factors influencing motivation and work engagement of healthcare professionals. Materia Socio-Med. 2022;34(3):216. 10.5455/msm.2022.34.216-224 PMC9559882 36310751

[33] Kaldal MH, Conroy T, Feo R, Grønkjær M, Voldbjerg SL. Umbrella review: newly graduated nurses’ experiences of providing direct care in hospital settings. J Adv Nurs. 2023;79(6):2058–69. 10.1111/jan.15434. 36070096 10.1111/jan.15434

[34] Jester R. Editorial – Global shortage of nurses – Rebecca Jester for May 2023 issue. Int J Orthop Trauma Nurs. 2023;49:101018. 10.1016/j.ijotn.2023.101018. 37041090 10.1016/j.ijotn.2023.101018 PMC10038672

[35] Holá J, Mazouch P, Fischer J, et al. Planning the optimal general nurse graduates: methodology and significance. Discov Health Syst. 2024;3:90. 10.1007/s44250-024-00155-w.

[36] Aiken LH, et al. Nurse staffing and education and hospital mortality in nine European countries: a retrospective observational study. Lancet. 2014;383(9931):1987–98. 10.1016/S0140-6736(13)62631-8. 10.1016/S0140-6736(13)62631-8 PMC4035380 24581683

[37] Shaffer FA, Bakhshi M, Cook K, Álvarez TD. International Nurse Recruitment Beyond the COVID-19 Pandemic: Considerations for the Nursing Workforce Leader. Nurse Lead. 2022;20(2). 10.1016/j.mnl.2021.12.001. 10.1016/j.mnl.2021.12.001 PMC8850103 35194412

[38] Ministry of Health of the Czech Republic. Almost 13 billion will be invested in healthcare. A new program will significantly increase the educational capacity for non-physician professions [Do zdravotnictví zamíří investice téměř 13 miliard. Nový program výrazně navýší kapacity vzdělávání nelékařských profesí]. Press conference. Praha: Ministerstvo zdravotnictví ČR. 2025. https://mzd.gov.cz/tiskove-centrum-mz/do-zdravotnictvi-zamiri-investice-temer-13-miliard-novy-program-vyrazne-navysi-kapacity-vzdelavani-nelekarskych-profesi/. Accessed 16. 8. 2025. Czech.

[39] Ha TTT, Thuy LT, Thanh DTH. Factors affecting career turnover intention after graduation among nursing students: a cross-sectional study in Central Vietnam. Nurs Pract Today. 2023;10:229–38. 10.18502/npt.v10i3.13432.

[40] Lin Y, Hu Z, Danaee M, Alias H, Wong LP. The impact of the COVID-19 pandemic on future nursing career turnover intention among nursing students. Risk Manag Healthc Policy. 2021;14:3605–15. 10.2147/RMHP.S321855. 34475792 10.2147/RMHP.S322764 PMC8407786

[41] Cao X, Li J, Gong S. Effects of resilience, social support, and work environment on turnover intention in newly graduated nurses: the mediating role of transition shock. J Nurs Adm Manag. 2021;29(8):2585–93. 10.1111/jonm.13418. 10.1111/jonm.13418 34252240

[42] Kadefors R, Nilsson K, Rylander L, Östergren P-O, Albin M. Occupation, gender and work-life exits: a Swedish population study. Ageing Soc. 2018;38(7):1332–49. 10.1017/S0144686X17000083.

[43] ALobaid AM, Gosling CM, Khasawneh E, McKenna L, Williams B. Challenges Faced by Female Healthcare Professionals in the Workforce: A Scoping Review. J Multidiscip Healthc. 2020;13:681–91. 32821112 10.2147/JMDH.S254922 PMC7417925

